# Tetra­kis­(μ-benzoato-κ^2^ *O*:*O*′)bis{[4-(di­methyl­amino)­pyridine-κ*N* ^1^]zinc(II)}

**DOI:** 10.1107/S1600536811001188

**Published:** 2011-01-15

**Authors:** Zhe-Yin Yu, Kun-Hua Lin, Fei-Fei Zhang, Min Shao, Ming Li

**Affiliations:** a State Key Laboratory of Metal Matrix Composites, School of Materials Science and Engineering, Shanghai Jiao Tong University, Shanghai 200240, People’s Republic of China; b Department of Chemistry, College of Science, Shanghai University, Shanghai 200444, People’s Republic of China; c Instrumental Analysis and Research Center, Shanghai University, Shanghai 200444, People’s Republic of China

## Abstract

In the centrosymmetric binuclear title complex, [Zn_2_(C_7_H_5_O_2_)_4_(C_7_H_10_N_2_)_2_], the Zn atoms [Zn⋯Zn = 3.0037 (6) Å] are bridged by four benzoate ligands. Each of the Zn atoms assumes an approximately square-pyramidal environment, with four O atoms in a plane and the pyridine N atom at the apical site.

## Related literature

For the nucleophilic properties of 4-(dimethyl­amino)­pyridine (DMAP), see: Fu (2000 ▶). For complexes of DMAP, see: Tyrra *et al.* (2003 ▶) and for complexes of DMAP which exhibit luminescence, see: Araki *et al.* (2005 ▶). For Zn⋯Zn distances in related structures, see: Anirban *et al.* (2006 ▶); Han *et al.* (2009 ▶); Konidaris *et al.* (2009 ▶); Wang *et al.* (2008 ▶). 
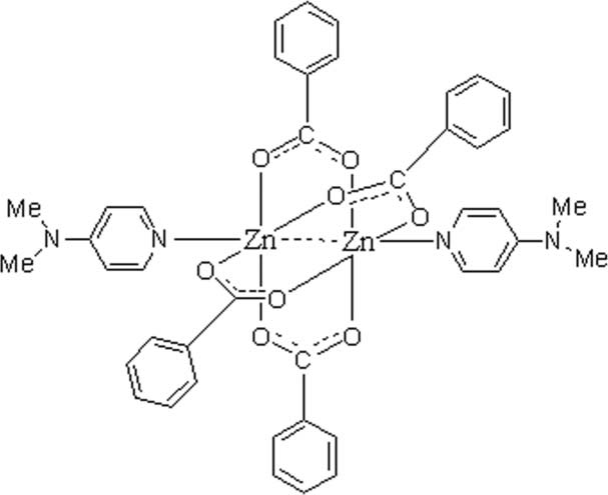


## Experimental

### 

#### Crystal data

[Zn_2_(C_7_H_5_O_2_)_4_(C_7_H_10_N_2_)_2_]
*M* *_r_* = 859.56
Monoclinic, 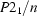

*a* = 10.3146 (12) Å
*b* = 11.1558 (13) Å
*c* = 17.324 (2) Å
β = 95.616 (1)°
*V* = 1983.9 (4) Å^3^
*Z* = 2
Mo *K*α radiation
μ = 1.27 mm^−1^
*T* = 296 K
0.30 × 0.30 × 0.20 mm

#### Data collection

Bruker SMART CCD area-detector diffractometer
Absorption correction: multi-scan (*SADABS*; Sheldrick, 1996 ▶) *T*_min_ = 0.702, *T*_max_ = 0.786
10123 measured reflections
3515 independent reflections
2860 reflections with *I* > 2σ(*I*)
*R*_int_ = 0.030

#### Refinement

*R*[*F* ^2^ > 2σ(*F* ^2^)] = 0.031
*wR*(*F* ^2^) = 0.066
*S* = 0.99
3515 reflections
255 parameters
H-atom parameters constrained
Δρ_max_ = 0.27 e Å^−3^
Δρ_min_ = −0.20 e Å^−3^

### 

Data collection: *SMART* (Bruker, 2000 ▶); cell refinement: *SAINT* (Bruker, 2000 ▶); data reduction: *SAINT*; program(s) used to solve structure: *SHELXS97* (Sheldrick, 2008 ▶); program(s) used to refine structure: *SHELXL97* (Sheldrick, 2008 ▶); molecular graphics: *SHELXTL* (Sheldrick, 2008 ▶); software used to prepare material for publication: *SHELXTL*.

## Supplementary Material

Crystal structure: contains datablocks global, I. DOI: 10.1107/S1600536811001188/bq2273sup1.cif

Structure factors: contains datablocks I. DOI: 10.1107/S1600536811001188/bq2273Isup2.hkl

Additional supplementary materials:  crystallographic information; 3D view; checkCIF report

## Figures and Tables

**Table 1:** Selected bond lengths (Å)

Zn1—N1	2.0134 (19)
Zn1—O3	2.0390 (18)
Zn1—O1	2.0467 (16)
Zn1—O2^i^	2.0472 (17)
Zn1—O4^i^	2.0782 (18)

Symmetry code: (i) 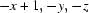.

**Fig. 1.**
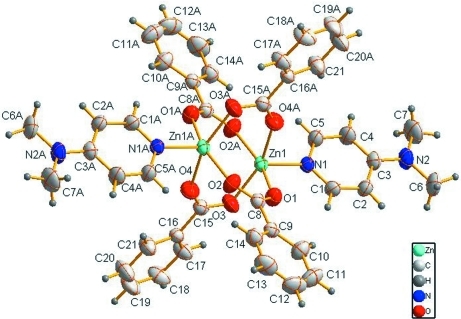
Molecular structure of the title compound with displacement ellipsoids drawn at the 50% probability level. Symmetry code for atoms labeled with A : -x, -y, -z

## supplementary crystallographic information

### Comment

4-(dimethylamino)pyridine (DMAP) has good coordination ability, but there are few reports on its complexes (Tyrra *et al.*, 2003) except a lot of reports on its nucleophilic properties (Fu *et al.*, 2000). The DMAP complexes which exhibit luminescence properties was reported (Araki *et al.*, 2005). In our systematic studies on transition metal complexes with the DMAP, the title compound was prepared and its x-ray structure is presented here.

The title compound has a paddlewheel type dimeric structure (Fig. 1) with an inversion centre located between the Zn ions. Each Zn atom is five-coordinate: the four equatorial O atoms belong to four bridging carboxyl groups and the N atom at the apical position is the pyridine N atom of the DMAP ligand. The Zn—O bond lengths are between 2.0390 Å and 2.0782 Å, but the Zn1—N1 distance is shorter [2.0134 (19) Å] (Table 1.). The Zn···Zn distance of 3.0037 (6) Å is longer than the corresponding distance of 2.9827 (7) Å in [Zn_2_(C_6_H_5_COO)_4_(H_2_O)_2_][Zn(C_6_H_5_COO)_2_(1,2-bis(4-pyridyl)ethane)] (Han *et al.*, 2009) and 2.9692 (15) Å in [Zn_2_ (C_6_H_5_COO)_2_(4,4'-bipyridine)]_n_ (Wang *et al.*, 2008) and 2.9582 (4) Å in [Zn_2_ (C_6_H_5_COO)_2_(pyridine)_2_] (Anirban *et al.*, 2006), but shorter than the corresponding distance of 3.392 (5) Å in {Zn_3_(C_6_H_5_COO)_6_[(3-py)(CH) NOH]_2_} (Konidaris *et al.*, 2009).

### Experimental

An ethanol solution (2 ml) containing DMAP (0.0611 g, 0.5 mmol) and Zn(CH_3_COO)_2_.2H_2_O (0.0549 g, 0.25 mmol) was mixed with an aqueous solution (5 ml) of benzoic acid (0.0611 g, 0.5 mmol) and NaOH (0.0200 g, 0.5 mmol). The mixture was refluxed for 5 h. The solution was filtered after cooling to room temperature. Colorless single crystals suitable for x-ray diffraction were obtained from the filtrate after 6 days.

### Refinement

Methyl H atoms were placed in calculated positions with C—H distances = 0.96 Å and *U*_iso_(H) = 1.5*U*_eq_(C). Other H atoms were placed in calculated positions with C—H distances = 0.93 Å and *U*_iso_(H) = 1.2*U*_eq_(C).

### Crystal data

**Table:**

[Zn_2_(C_7_H_5_O_2_)_4_(C_7_H_10_N_2_)_2_]	*F*(000) = 888
*M**_r_* = 859.56	*D*_x_ = 1.439 Mg m^−^^3^
Monoclinic, *P*2_1_/*n*	Mo *K*α radiation, λ = 0.71073 Å
Hall symbol: -P 2yn	Cell parameters from 3350 reflections
*a* = 10.3146 (12) Å	θ = 2.4–24.2°
*b* = 11.1558 (13) Å	µ = 1.27 mm^−^^1^
*c* = 17.324 (2) Å	*T* = 296 K
β = 95.616 (1)°	Block, colorless
*V* = 1983.9 (4) Å^3^	0.30 × 0.30 × 0.20 mm
*Z* = 2	

### Data collection

**Table:**

Bruker SMART CCD area-detector diffractometer	3515 independent reflections
Radiation source: fine-focus sealed tube	2860 reflections with *I* > 2σ(*I*)
graphite	*R*_int_ = 0.030
φ and ω scans	θ_max_ = 25.1°, θ_min_ = 2.4°
Absorption correction: multi-scan (*SADABS*; Sheldrick, 1996)	*h* = −12→12
*T*_min_ = 0.702, *T*_max_ = 0.786	*k* = −9→13
10123 measured reflections	*l* = −20→20

### Refinement

**Table:**

Refinement on *F*^2^	Primary atom site location: structure-invariant direct methods
Least-squares matrix: full	Secondary atom site location: difference Fourier map
*R*[*F*^2^ > 2σ(*F*^2^)] = 0.031	Hydrogen site location: inferred from neighbouring sites
*wR*(*F*^2^) = 0.066	H-atom parameters constrained
*S* = 0.99	*w* = 1/[σ^2^(*F*_o_^2^) + (0.0188*P*)^2^ + 1.4303*P*] where *P* = (*F*_o_^2^ + 2*F*_c_^2^)/3
3515 reflections	(Δ/σ)_max_ < 0.001
255 parameters	Δρ_max_ = 0.27 e Å^−^^3^
0 restraints	Δρ_min_ = −0.20 e Å^−^^3^

### Special details

**Table:**

Geometry. All e.s.d.'s (except the e.s.d. in the dihedral angle between two l.s. planes) are estimated using the full covariance matrix. The cell e.s.d.'s are taken into account individually in the estimation of e.s.d.'s in distances, angles and torsion angles; correlations between e.s.d.'s in cell parameters are only used when they are defined by crystal symmetry. An approximate (isotropic) treatment of cell e.s.d.'s is used for estimating e.s.d.'s involving l.s. planes.
Refinement. Refinement of *F*^2^ against ALL reflections. The weighted *R*-factor *wR* and goodness of fit *S* are based on *F*^2^, conventional *R*-factors *R* are based on *F*, with *F* set to zero for negative *F*^2^. The threshold expression of *F*^2^ > σ(*F*^2^) is used only for calculating *R*-factors(gt) *etc*. and is not relevant to the choice of reflections for refinement. *R*-factors based on *F*^2^ are statistically about twice as large as those based on *F*, and *R*- factors based on ALL data will be even larger.

### Fractional atomic coordinates and isotropic or equivalent isotropic displacement parameters (Å^2^)

**Table:**

	*x*	*y*	*z*	*U*_iso_*/*U*_eq_	
Zn1	0.63121 (3)	−0.05006 (2)	0.028407 (16)	0.03192 (9)	
C1	0.8975 (2)	−0.1390 (2)	0.00916 (15)	0.0405 (6)	
H1	0.9011	−0.0714	−0.0220	0.049*	
C2	1.0020 (2)	−0.2142 (2)	0.01489 (15)	0.0445 (7)	
H2	1.0739	−0.1969	−0.0117	0.053*	
C3	1.0015 (2)	−0.3180 (2)	0.06093 (15)	0.0412 (6)	
C4	0.8891 (3)	−0.3349 (2)	0.09862 (16)	0.0504 (7)	
H4	0.8824	−0.4014	0.1303	0.060*	
C5	0.7892 (3)	−0.2544 (2)	0.08927 (16)	0.0478 (7)	
H5	0.7159	−0.2691	0.1150	0.057*	
C6	1.2169 (3)	−0.3787 (3)	0.0269 (2)	0.0689 (9)	
H6A	1.1904	−0.3655	−0.0271	0.103*	
H6B	1.2711	−0.4487	0.0326	0.103*	
H6C	1.2649	−0.3104	0.0477	0.103*	
C7	1.1030 (3)	−0.4980 (3)	0.1208 (2)	0.0769 (11)	
H7A	1.0949	−0.4700	0.1725	0.115*	
H7B	1.1833	−0.5412	0.1198	0.115*	
H7C	1.0312	−0.5499	0.1047	0.115*	
C8	0.5952 (2)	0.0937 (2)	−0.11840 (14)	0.0352 (6)	
C9	0.6546 (2)	0.1572 (2)	−0.18291 (13)	0.0356 (6)	
C10	0.7862 (3)	0.1480 (3)	−0.19036 (16)	0.0515 (7)	
H10	0.8382	0.0994	−0.1564	0.062*	
C11	0.8415 (3)	0.2103 (3)	−0.24778 (18)	0.0687 (9)	
H11	0.9303	0.2033	−0.2524	0.082*	
C12	0.7658 (4)	0.2825 (3)	−0.29808 (19)	0.0721 (10)	
H12	0.8032	0.3253	−0.3363	0.086*	
C13	0.6348 (4)	0.2911 (3)	−0.29167 (17)	0.0670 (9)	
H13	0.5829	0.3389	−0.3262	0.080*	
C14	0.5794 (3)	0.2289 (2)	−0.23407 (15)	0.0489 (7)	
H14	0.4905	0.2357	−0.2299	0.059*	
C15	0.5854 (3)	0.1832 (2)	0.08035 (14)	0.0388 (6)	
C16	0.6088 (2)	0.2987 (2)	0.12389 (14)	0.0359 (6)	
C17	0.7090 (3)	0.3097 (2)	0.18238 (16)	0.0513 (7)	
H17	0.7658	0.2459	0.1933	0.062*	
C18	0.7261 (3)	0.4143 (3)	0.22497 (17)	0.0609 (8)	
H18	0.7924	0.4202	0.2652	0.073*	
C19	0.6448 (3)	0.5083 (3)	0.2074 (2)	0.0686 (9)	
H19	0.6551	0.5785	0.2362	0.082*	
C20	0.5481 (3)	0.5007 (3)	0.1477 (2)	0.0739 (10)	
H20	0.4950	0.5667	0.1351	0.089*	
C21	0.5290 (3)	0.3959 (3)	0.10635 (18)	0.0550 (8)	
H21	0.4621	0.3906	0.0664	0.066*	
N1	0.78988 (18)	−0.15549 (18)	0.04532 (11)	0.0354 (5)	
N2	1.1022 (2)	−0.3960 (2)	0.06836 (14)	0.0556 (6)	
O1	0.67051 (17)	0.03548 (16)	−0.07120 (10)	0.0453 (5)	
O2	0.47447 (17)	0.10480 (17)	−0.11606 (10)	0.0469 (5)	
O3	0.67429 (19)	0.10512 (16)	0.08761 (11)	0.0518 (5)	
O4	0.47960 (18)	0.17275 (17)	0.04000 (11)	0.0527 (5)	

### Atomic displacement parameters (Å^2^)

**Table:**

	*U*^11^	*U*^22^	*U*^33^	*U*^12^	*U*^13^	*U*^23^
Zn1	0.02949 (15)	0.03168 (16)	0.03423 (16)	0.00493 (12)	0.00125 (11)	−0.00011 (13)
C1	0.0364 (14)	0.0381 (15)	0.0468 (15)	0.0033 (11)	0.0032 (12)	0.0100 (12)
C2	0.0336 (14)	0.0471 (16)	0.0540 (17)	0.0053 (12)	0.0112 (12)	0.0081 (14)
C3	0.0379 (15)	0.0423 (16)	0.0426 (15)	0.0095 (12)	0.0006 (12)	0.0009 (12)
C4	0.0529 (17)	0.0440 (17)	0.0560 (17)	0.0136 (14)	0.0140 (14)	0.0212 (14)
C5	0.0425 (16)	0.0479 (18)	0.0554 (17)	0.0078 (13)	0.0165 (13)	0.0117 (14)
C6	0.0460 (18)	0.070 (2)	0.092 (3)	0.0233 (16)	0.0155 (17)	−0.0007 (19)
C7	0.078 (2)	0.058 (2)	0.094 (3)	0.0313 (18)	0.003 (2)	0.021 (2)
C8	0.0403 (15)	0.0335 (14)	0.0323 (13)	−0.0005 (11)	0.0054 (12)	−0.0039 (11)
C9	0.0431 (15)	0.0335 (14)	0.0309 (13)	−0.0023 (11)	0.0071 (11)	−0.0032 (11)
C10	0.0463 (17)	0.065 (2)	0.0439 (16)	−0.0012 (14)	0.0082 (13)	0.0045 (14)
C11	0.059 (2)	0.091 (3)	0.060 (2)	−0.0180 (19)	0.0240 (17)	−0.0015 (19)
C12	0.099 (3)	0.068 (2)	0.054 (2)	−0.024 (2)	0.029 (2)	0.0061 (17)
C13	0.099 (3)	0.053 (2)	0.0504 (19)	0.0054 (19)	0.0109 (18)	0.0174 (15)
C14	0.0572 (18)	0.0435 (17)	0.0468 (16)	0.0054 (14)	0.0098 (14)	0.0045 (13)
C15	0.0478 (16)	0.0354 (15)	0.0343 (14)	−0.0030 (12)	0.0100 (13)	−0.0027 (11)
C16	0.0393 (14)	0.0309 (14)	0.0375 (14)	−0.0026 (11)	0.0047 (11)	−0.0037 (11)
C17	0.0591 (18)	0.0380 (16)	0.0534 (17)	0.0031 (13)	−0.0116 (14)	−0.0005 (13)
C18	0.072 (2)	0.050 (2)	0.0552 (19)	−0.0074 (16)	−0.0186 (16)	−0.0095 (15)
C19	0.078 (2)	0.0427 (19)	0.083 (2)	−0.0012 (17)	−0.005 (2)	−0.0268 (17)
C20	0.068 (2)	0.0414 (18)	0.108 (3)	0.0141 (16)	−0.015 (2)	−0.0204 (19)
C21	0.0503 (17)	0.0445 (18)	0.067 (2)	0.0064 (14)	−0.0091 (15)	−0.0137 (15)
N1	0.0320 (11)	0.0343 (12)	0.0397 (12)	0.0057 (9)	0.0031 (9)	0.0040 (9)
N2	0.0487 (14)	0.0509 (15)	0.0678 (16)	0.0235 (12)	0.0091 (12)	0.0100 (13)
O1	0.0433 (10)	0.0516 (12)	0.0418 (10)	0.0097 (9)	0.0076 (8)	0.0137 (9)
O2	0.0357 (10)	0.0635 (13)	0.0419 (10)	0.0024 (9)	0.0070 (8)	0.0109 (9)
O3	0.0574 (12)	0.0360 (11)	0.0605 (12)	0.0055 (9)	−0.0017 (10)	−0.0124 (9)
O4	0.0491 (12)	0.0513 (12)	0.0553 (12)	−0.0048 (9)	−0.0062 (10)	−0.0180 (9)

### Geometric parameters (Å, °)

**Table:**

Zn1—N1	2.0134 (19)	C9—C14	1.376 (3)
Zn1—O3	2.0390 (18)	C9—C10	1.380 (3)
Zn1—O1	2.0467 (16)	C10—C11	1.381 (4)
Zn1—O2^i^	2.0472 (17)	C10—H10	0.9300
Zn1—O4^i^	2.0782 (18)	C11—C12	1.373 (5)
Zn1—Zn1^i^	3.0037 (6)	C11—H11	0.9300
C1—N1	1.340 (3)	C12—C13	1.371 (5)
C1—C2	1.362 (3)	C12—H12	0.9300
C1—H1	0.9300	C13—C14	1.384 (4)
C2—C3	1.406 (4)	C13—H13	0.9300
C2—H2	0.9300	C14—H14	0.9300
C3—N2	1.351 (3)	C15—O4	1.243 (3)
C3—C4	1.398 (4)	C15—O3	1.262 (3)
C4—C5	1.364 (3)	C15—C16	1.500 (3)
C4—H4	0.9300	C16—C17	1.380 (3)
C5—N1	1.341 (3)	C16—C21	1.377 (4)
C5—H5	0.9300	C17—C18	1.382 (4)
C6—N2	1.456 (4)	C17—H17	0.9300
C6—H6A	0.9600	C18—C19	1.358 (4)
C6—H6B	0.9600	C18—H18	0.9300
C6—H6C	0.9600	C19—C20	1.367 (4)
C7—N2	1.456 (4)	C19—H19	0.9300
C7—H7A	0.9600	C20—C21	1.376 (4)
C7—H7B	0.9600	C20—H20	0.9300
C7—H7C	0.9600	C21—H21	0.9300
C8—O1	1.253 (3)	O2—Zn1^i^	2.0471 (17)
C8—O2	1.256 (3)	O4—Zn1^i^	2.0782 (18)
C8—C9	1.503 (3)		
			
N1—Zn1—O3	106.79 (8)	C10—C9—C8	120.7 (2)
N1—Zn1—O1	99.78 (7)	C9—C10—C11	120.6 (3)
O3—Zn1—O1	88.67 (8)	C9—C10—H10	119.7
N1—Zn1—O2^i^	101.66 (8)	C11—C10—H10	119.7
O3—Zn1—O2^i^	89.18 (8)	C12—C11—C10	120.2 (3)
O1—Zn1—O2^i^	158.15 (7)	C12—C11—H11	119.9
N1—Zn1—O4^i^	95.29 (8)	C10—C11—H11	119.9
O3—Zn1—O4^i^	157.92 (8)	C13—C12—C11	119.6 (3)
O1—Zn1—O4^i^	88.25 (8)	C13—C12—H12	120.2
O2^i^—Zn1—O4^i^	85.61 (8)	C11—C12—H12	120.2
N1—Zn1—Zn1^i^	163.45 (6)	C12—C13—C14	120.2 (3)
O3—Zn1—Zn1^i^	89.66 (5)	C12—C13—H13	119.9
O1—Zn1—Zn1^i^	78.28 (5)	C14—C13—H13	119.9
O2^i^—Zn1—Zn1^i^	79.97 (5)	C9—C14—C13	120.7 (3)
O4^i^—Zn1—Zn1^i^	68.30 (5)	C9—C14—H14	119.7
N1—C1—C2	124.6 (2)	C13—C14—H14	119.7
N1—C1—H1	117.7	O4—C15—O3	125.5 (2)
C2—C1—H1	117.7	O4—C15—C16	116.9 (2)
C1—C2—C3	120.1 (2)	O3—C15—C16	117.6 (2)
C1—C2—H2	119.9	C17—C16—C21	118.7 (2)
C3—C2—H2	119.9	C17—C16—C15	121.2 (2)
N2—C3—C4	122.2 (2)	C21—C16—C15	120.1 (2)
N2—C3—C2	122.6 (2)	C16—C17—C18	121.0 (3)
C4—C3—C2	115.2 (2)	C16—C17—H17	119.5
C5—C4—C3	120.5 (2)	C18—C17—H17	119.5
C5—C4—H4	119.8	C19—C18—C17	119.2 (3)
C3—C4—H4	119.8	C19—C18—H18	120.4
N1—C5—C4	124.3 (2)	C17—C18—H18	120.4
N1—C5—H5	117.9	C18—C19—C20	120.7 (3)
C4—C5—H5	117.9	C18—C19—H19	119.6
N2—C6—H6A	109.5	C20—C19—H19	119.6
N2—C6—H6B	109.5	C19—C20—C21	120.2 (3)
H6A—C6—H6B	109.5	C19—C20—H20	119.9
N2—C6—H6C	109.5	C21—C20—H20	119.9
H6A—C6—H6C	109.5	C20—C21—C16	120.2 (3)
H6B—C6—H6C	109.5	C20—C21—H21	119.9
N2—C7—H7A	109.5	C16—C21—H21	119.9
N2—C7—H7B	109.5	C1—N1—C5	115.4 (2)
H7A—C7—H7B	109.5	C1—N1—Zn1	123.41 (16)
N2—C7—H7C	109.5	C5—N1—Zn1	120.93 (16)
H7A—C7—H7C	109.5	C3—N2—C6	121.4 (2)
H7B—C7—H7C	109.5	C3—N2—C7	121.3 (2)
O1—C8—O2	125.7 (2)	C6—N2—C7	117.2 (2)
O1—C8—C9	117.2 (2)	C8—O1—Zn1	129.10 (16)
O2—C8—C9	117.1 (2)	C8—O2—Zn1^i^	126.60 (16)
C14—C9—C10	118.7 (2)	C15—O3—Zn1	114.55 (17)
C14—C9—C8	120.5 (2)	C15—O4—Zn1^i^	141.85 (18)

Symmetry codes: (i) −*x*+1, −*y*, −*z*.
